# Stereotaxic Coordinates of Human Hypothalamic Nuclei Used for Region of Interest Analyses in Functional Magnetic Resonance Imaging

**DOI:** 10.14789/jmj.JMJ24-0009-P

**Published:** 2024-04-18

**Authors:** NATSUKI OMORI RISON, AKITOSHI OGAWA, TAKAHIRO OSADA, SEIKI KONISHI

**Affiliations:** 1 Department of Clinical and Biomedical Sciences, University of Exeter Medical School, Exeter, UK; 1 Department of Clinical and Biomedical Sciences, University of Exeter Medical School, Exeter, UK; 2 Department of Neurophysiology, Juntendo University School of Medicine, Tokyo, Japan; 2 Department of Neurophysiology, Juntendo University School of Medicine, Tokyo, Japan; 3 Research Institute for Diseases of Old Age, Juntendo University School of Medicine, Tokyo, Japan; 3 Research Institute for Diseases of Old Age, Juntendo University School of Medicine, Tokyo, Japan; 4 Sportology Center, Juntendo University School of Medicine, Tokyo, Japan; 4 Sportology Center, Juntendo University School of Medicine, Tokyo, Japan; 5 Advanced Research Institute for Health Science, Juntendo University School of Medicine, Tokyo, Japan; 5 Advanced Research Institute for Health Science, Juntendo University School of Medicine, Tokyo, Japan

**Keywords:** functional magnetic resonance imaging, human, hypothalamus

## Abstract

The hypothalamus maintains homeostasis by controlling various organs and the central nervous system, but analyzing the human hypothalamic nuclei is challenging. Our previous studies applied areal parcellation to high-resolution functional magnetic resonance imaging (fMRI) data to delineate hypothalamic nuclei boundaries. This article presents stereotaxic coordinates of these nuclei for fMRI analyses, offering guidance on defining regions of interest and appropriate spatial smoothing kernel sizes. The provided coordinates aid future research in nuclear level hypothalamus analyses.

The hypothalamus can be considered as a functional center of the human body that maintains homeostasis by controlling various organs and the central nervous system. The hypothalamus contains lots of nuclei that play distinct functional roles, so it is essential to analyze these nuclei separately. However, separating these nuclei embedded within a small structure is challenging using noninvasive neuroimaging in humans. To resolve this issue, in our previous studies^1-6)^, we have applied areal parcellation^7-9)^ to high-resolution functional magnetic resonance imaging (fMRI) data to delineate the boundaries between these hypothalamic nuclei. Since we have covered most of the hypothalamic nuclei in our studies, it seems now worthwhile to summarize the published data and propose practical tools for fMRI analyses of the nuclei. Specifically, in this article, we present stereotaxic coordinates of these nuclei that can be used to define the regions of interest (ROIs) for the nuclei in spatial resolutions of 1.25 mm and 2 mm. We also propose an appropriate kernel size of spatial smoothing that maximizes the signal to noise ratio with minimal unwanted signal contamination.

Table 1 shows the stereotaxic coordinates of the human hypothalamic nuclei, namely the medial preoptic nucleus (MPO), suprachiasmatic nucleus (SCN), supraoptic nucleus (SO), paraventricular nucleus of the hypothalamus (PVH), anterior nucleus of the hypothalamus (AH), ventromedial nucleus of the hypothalamus (VMH), dorsomedial nucleus of the hypothalamus (DMH), arcuate nucleus (ARC), posterior nucleus of the hypothalamus (PH). The mammillary body is not included here since it is structurally evident. The lateral hypothalamic area (LHA) is not included either as it is relatively large. Thus, the listed nuclei are located in the medial part of the hypothalamus and can be approximately defined as X < 3 or X > -3, excluding the middle line ventricular voxels, of the hypothalamus.

**Table 1:** List of MNI coordinates of the hypothalamic nuclei

Resolution	1.25 mm isotropic			2.0 mm isotropic		
Nucleus	x	y	z	x	y	z
L MPO	-1.5	+2.3	-12.8	-2.0	+2.0	-12.0
R MPO	+1.5	+2.3	-12.8	+2.0	+2.0	-12.0
L SCN	-1.8	+1.5	-15.8	-2.0	+2.0	-16.0
R SCN	+1.8	+1.5	-15.8	+2.0	+2.0	-16.0
L SO	-5.5	+1.8	-12.8	-6.0	+2.0	-12.0
R SO	+4.3	+1.0	-12.5	+4.0	+2.0	-12.0
L PVH	-1.8	-0.5	-10.5	-2.0	±0.0	-10.0
R PVH	+1.5	+0.3	-10.8	+2.0	±0.0	-10.0
L AH	-2.0	-1.5	-18.0	-2.0	-2.0	-18.0
R AH	+1.8	-1.5	-18.0	+2.0	-2.0	-18.0
L VMH	-1.5	-4.1	-14.3	-2.0	-4.0	-14.0
R VMH	+1.3	-4.3	-14.2	+2.0	-4.0	-14.0
L DMH	-1.5	-4.3	-11.2	-2.0	-4.0	-12.0
R DMH	+1.5	-4.5	-10.8	+2.0	-4.0	-10.0
L ARC	-2.3	-4.8	-17.0	-2.0	-4.0	-18.0
R ARC	+2.3	-4.8	-17.0	+2.0	-4.0	-18.0
L PH	-1.5	-8.3	-8.3	-2.0	-8.0	-8.0
R PH	+1.5	-8.3	-8.3	+2.0	-8.0	-8.0

L = left, R = right, MPO = medial preoptic nucleus, SCN = suprachiasmatic nucleus, SO = supraoptic nucleus, PVH = paraventricular nucleus of the hypothalamus, AH = anterior nucleus of the hypothalamus, VMH = ventromedial nucleus of the hypothalamus, DMH = dorsomedial nucleus of the hypothalamus, ARC = arcuate nucleus, PH = posterior nucleus of the hypothalamus.

In the 1.25 mm resolution, the coordinates are the centroids of multi-voxel ROIs for the hypothalamic nuclei calculated based on the areal parcellation method. Although it is possible to use the coordinates for your ROI analyses, it would be more appropriate to use the multi-voxel ROI. The ROI files can be found at the dryad website (https://doi.org/10.5061/dryad.6q573n620) or please contact us for details. The Gaussian smoothing kernel could be 4 mm in full width at half maximum, in either single-voxel or multi-voxel ROIs (see Discussion in Ogawa et al., 2022^4)^ for validation). However, the SCN is one exception in the list, and the smoothing kernel should be 2 mm because of its smaller size (see Discussion in Oka et al., 2024^6)^ for validation). In the 2 mm resolution, as the size of each voxel is four times larger, it is appropriate to use the single voxels listed in the table as ROIs, and the smoothing kernel size should be set the same as previously mentioned at 4 mm in all nuclei except for the SCN (2 mm). When scanning original image data, the image resolution should be at 1.25 mm to make use of the 1.25 mm analysis. However, if scanning at this resolution is not possible or a large database taken in 2 mm resolution is utilized, the 2 mm analysis may be used. We hope this guidance is helpful to future followers of the nuclear level analyses of the hypothalamus.

## Funding

No funding was received.

## Author contributions

All authors read and approved the final manuscript.

## Conflicts of interest statement

The authors declare that there are no conflicts of interest.
